# AI in respiratory care: findings from the GOLD report

**DOI:** 10.1186/s12967-026-08454-x

**Published:** 2026-06-13

**Authors:** Ali Al-waqeerah, Ahmed Bashah, Eslam Ghaleb, Huanyu He, Lili Gao

**Affiliations:** 1 Department of Respiratory Medicine, The First Affiliated Hospital of Dalian Medical University, Dalian, 116011 People’s Republic of China; 2 Department of Stomatology, The First Affiliated Hospital of Dalian Medical University, Dalian, 116011 People’s Republic of China; 3 College of Basic Medical Science, Dalian Medical University, Dalian, 116044 People’s Republic of China

**Keywords:** COPD, 2025 GOLD report, LLMs, ChatGPT-5, DeepSeek-V3.2, Gemini 3, Grok, Manus, Kimi K1.5, Artificial intelligence

## Abstract

**Background:**

Chronic obstructive pulmonary disease (COPD) requires personalised, guideline-based management according to the Global Initiative for Chronic Obstructive Lung Disease (GOLD) Report. As large language models (LLMs) are increasingly used for medical guidance, their adherence to updated clinical recommendations requires systematic evaluation. This study assessed and compared ChatGPT-5, DeepSeek-V3.2, Gemini 3, Grok, Manus, and Kimi K1.5 in delivering 2025 GOLD-consistent responses, focusing on accuracy, consistency, and clinical reasoning.

**Methods:**

A standardised dataset of 90 questions derived from the 2025 GOLD Report was categorised into YES/NO, multiple-choice, and open-ended formats. During a 7-day longitudinal study in December 2025, all questions were administered daily across the six platforms, generating 3,780 responses. Performance was evaluated against the 2025 GOLD Report, and open-ended responses were assessed for quality and clinical comprehensiveness.

**Results:**

Performance was assessed across two separate primary outcomes. For binary accuracy (YES/NO and MCQ questions), Kimi K1.5 achieved the highest rate (97.1%; 408/420; 95% CI, 95.1–98.4%), and Gemini 3 the lowest (89.5%; 376/420; 95% CI, 86.2–92.1%). For open-ended clinical reasoning quality, Gemini 3 achieved the highest weighted score (96.5%; 95% CI, 93.3–98.4%) and DeepSeek-V3.2 the lowest (91.8%; 95% CI, 87.4–94.9%), reflecting a performance inversion between the two outcome domains. All platforms exhibited statistical stability across the seven-day study period (*p* > 0.05 for all models).

**Conclusion:**

Large language models showed high but format-dependent adherence to the 2025 GOLD COPD guidelines, with clear divergence between binary accuracy and open-ended reasoning. While they may serve as supplementary tools under expert supervision, generalization to other settings requires further validation. Residual performance gaps persisted across both domains, with binary error rates ranging from 2.9% (Kimi K1.5) to 10.5% (Gemini 3), and open-ended weighted-score deficits ranging from 3.5% (Gemini 3) to 8.2% (DeepSeek-V3.2).

**Supplementary Information:**

The online version contains supplementary material available at 10.1186/s12967-026-08454-x.

## Introduction

Chronic obstructive pulmonary disease (COPD) is one of the leading causes of death and illness worldwide, and its prevalence is increasing as a public health concern [1, 2]. Damage to the airways or air sacs causes long-term breathing issues and poor airflow, which characterize this illness. Long-term exposure to dangerous particles or gases generally induces this damage [3, 4]. COPD is not only very common and deadly, but it also has a wide range of clinical characteristics, including different phenotypes, varying symptom burden, frequent comorbidities, and changing disease trajectories [5–7]. This heterogeneity makes it harder to diagnose, figure out the risk, and make treatment decisions; patient care needs to be tailored to each person while still following the same guidelines [8].

To address these challenges, evidence-based clinical practice guidelines play a central role in harmonizing COPD management. The Global Initiative for Chronic Obstructive Lung Disease (GOLD) has become the most trusted and frequently used system for diagnosing, evaluating, and treating COPD [9]. The 2025 GOLD Report incorporates new information on spirometric classification, the evaluation of symptom and flare-up risk, pharmacological and non-pharmacological treatments, and the management of comorbidities [10]. GOLD seeks to enhance clinical outcomes, lessen unnecessary variance in care, and facilitate decision-making in a variety of healthcare settings by offering organized, consensus-based recommendations. Therefore, following these rules is crucial for clinical practice as well as medical education.

Simultaneously, artificial intelligence (AI), especially large language model (LLM)-based systems, has rapidly emerged in recent years as tools that can synthesize vast amounts of medical information and produce solutions to clinical queries that are similar to those of a person [11]. AI large language models (LLMs) can handle a variety of inquiry types, such as multiple-choice, open-ended, and YES/NO [12–14]. Clinicians, trainees, and patients are increasingly using platforms like ChatGPT-5, DeepSeek-V3.2, Gemini 3, Grok, Manus, and Kimi K1.5 for disease information, diagnostic reasoning, and therapy recommendations [15–17]. These systems use transformer-based neural architectures that have been trained on large and diverse text corpora, allowing for quick information retrieval and context-aware answers. Because of this, LLMs have garnered a lot of attention as possible supplements for clinical decision support, the distribution of guidelines, and medical education [11, 18].

Recent advances in artificial intelligence, particularly large language models (LLMs), offer promising tools for improving the management of chronic respiratory diseases such as chronic obstructive pulmonary disease (COPD). LLMs can synthesize large volumes of medical literature and clinical data to support evidence-based decision-making, guideline interpretation, and clinical documentation, potentially enhancing adherence to clinical recommendations, assisting in patient education, enhancing early detection and monitoring strategies for chronic respiratory diseases, and improving efficiency in respiratory care [19–21].

Despite their many promises, the use of LLMs in clinical environments raises substantial concerns [22]. AI-generated medical information may contain errors, poor reasoning, a lack of transparency in source attribution, and unpredictability in replies across several queries [23, 24]. Guideline discordance is particularly critical when AI results diverge significantly or minimally from established evidence-based recommendations [25].

The purpose of this study is to systematically evaluate the accuracy, consistency, and adherence to guidelines of AI-generated responses to COPD management by comparing six large language models (ChatGPT-5, DeepSeek-V3.2, Gemini 3, Grok, Manus, and Kimi K1.5) to the 2025 GOLD report. Since the treatment of COPD is based on well-organised algorithms and particular pharmacological indications, unvalidated AI advice might lead to inaccurate treatment choices or an incorrect assessment of the severity of the illness. Using 90 standardized questions directly derived from the GOLD 2025 guideline provides an objective, reproducible framework for evaluating model performance across key domains, including diagnosis, assessment, pharmacologic and non-pharmacologic therapy, exacerbation management, and comorbidities, while minimizing bias and reflecting real-world clinical queries. The goal is to define AI’s current strengths and limitations as a supportive tool in respiratory medicine, while recognizing that it must supplement, not replace, clinician expertise and guideline-based care.

## Methods

### Study design and data collection

Between December 4 and 10, 2025, six artificial intelligence (AI) large language models (LLMs), ChatGPT-5, DeepSeek-V3.2, Gemini 3, Grok, Manus, and Kimi K1.5 were evaluated in a systematic, longitudinal manner. The study assessed the accuracy and consistency of their replies to clinical questions on the diagnosis and treatment of Chronic Obstructive Pulmonary Disease (COPD) in comparison to the 2025 Global Initiative for Chronic Obstructive Lung Disease (GOLD) Report.

### Question set development and validation

A list of 90 questions was methodically compiled from the 2025 GOLD Report. The questions were divided into three formats:

1. 30 YES/NO questions (Supplementary File 1).
2. 30 multiple-choice questions (MCQs) with a single best answer out of four alternatives (Supplementary File 2).
3. 30 open-ended questions that require explanations for diagnosis, therapy, exacerbation management, and follow-up (Supplementary File 3).

**Fig. 1:**
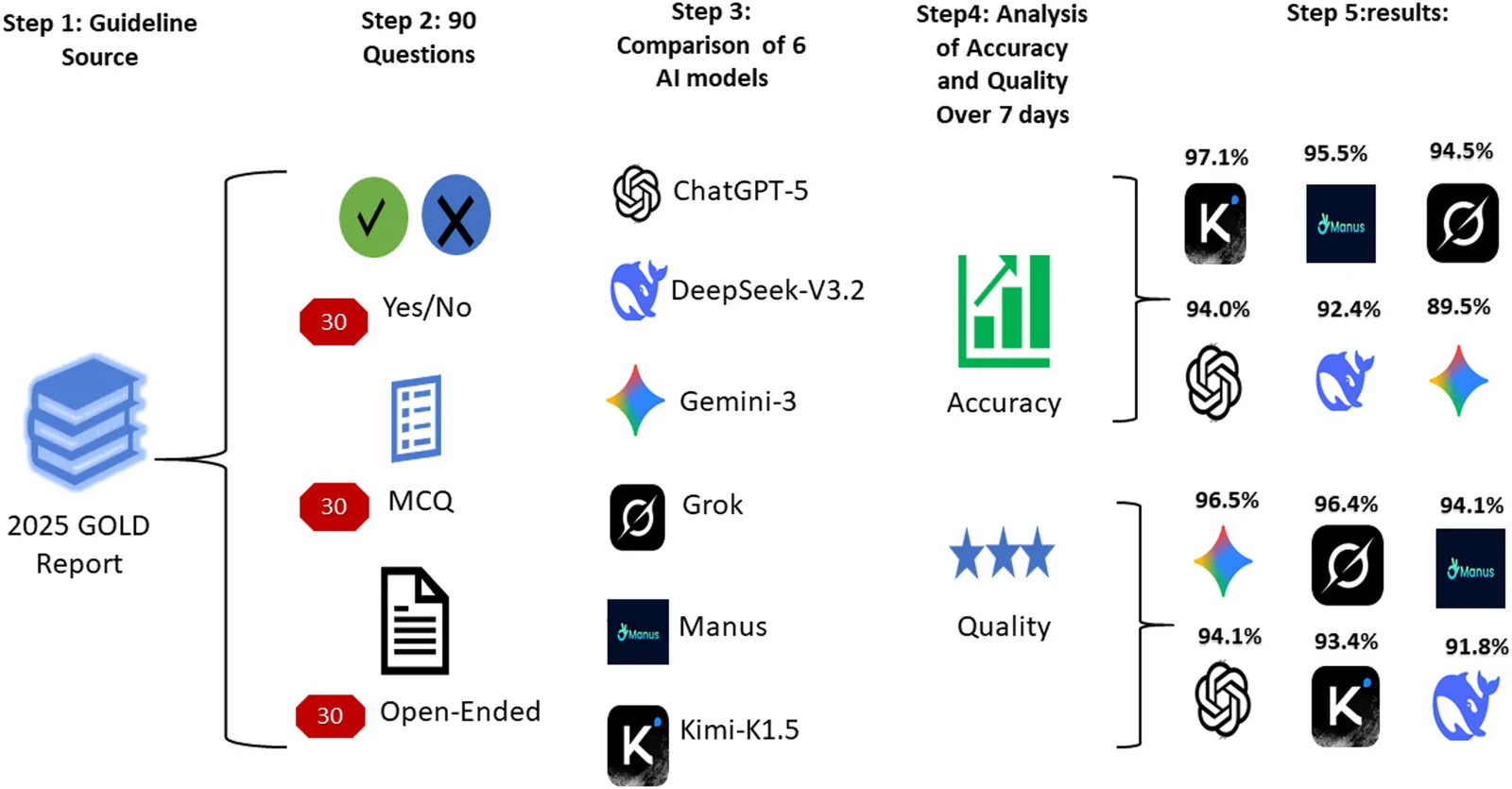
Flowchart illustrating the steps for assessing large model language responses

A final answer key was derived from the 2025 GOLD Report, and the questions were deliberately designed to ensure clinical relevance and comprehensive coverage of major COPD diagnostic and treatment areas, spanning six domains: spirometry and diagnosis, symptom and exacerbation risk assessment, pharmacological treatment, non-pharmacological management, exacerbation management, and comorbidities. The full question set and answer key were prepared before large language models (LLMs) testing and remained unchanged thereafter (Fig. 1).

Questions were developed by the first author (A.A.) with direct reference to the 2025 GOLD Report and reviewed for clinical accuracy by the senior pulmonologist co-author (L.G.), who verified each answer against the guideline. It should be noted that independent multi-annotator validation was not performed, which is a limitation of the benchmark instrument acknowledged in the Limitations section.

### Model selection and interaction protocol

During the study period (December 4–10, 2025), the following large language model (LLM) systems were assessed using their official web-based interfaces with the version identifiers displayed at the time of testing: ChatGPT-5 (https://chat.openai.com; displayed “GPT-5”), DeepSeek-V3.2 (https://chat.deepseek.com; displayed “DeepSeek-V3.2”), Google Gemini 3 (https://gemini.google.com/app; displayed “displayed ‘Gemini 3”), Grok (https://grok.com; no explicit version string was displayed in the interface), Manus (https://manus.im/app; no explicit version string was displayed; note: Manus is architecturally an autonomous AI agent rather than a conventional conversational LLM, and was evaluated here under single-turn, new-session constraints for comparability), and Kimi K1.5 (https://www.kimi.com; displayed “Kimi K1.5”). All models were tested using default settings without system-prompt modification, plugins, retrieval augmentation, or web browsing. Queries were submitted in text-only format exactly as written, with each question initiated in a new session to minimize contextual bias and prompt-engineering variability. Responses were recorded with timestamps. Multiple-choice and yes/no items were scored as correct or incorrect according to GOLD criteria [10]. Open-ended responses were graded as high quality (100% guideline adherence), moderate quality (70% adherence with omission of secondary details), or low quality (≤ 30% adherence, guideline discordance). To assess scoring consistency, a senior pulmonologist (L.G.) independently audited a blinded stratified random sample of 90 open-ended responses (15 per model; 7% of 1,260 total). Complete agreement was observed, with no adjudication required, although future studies should include formal kappa confidence intervals and multi-reviewer validation.

### Performance evaluation and scoring

To ensure transparent comparison, model performance was evaluated using two separate primary outcomes, each expressed on a standardized 0–1 scale. Binary Accuracy combined YES/NO and MCQ performance into a single dichotomous metric:

$$\begin{aligned}&\:\mathrm{Binary}\,\mathrm{Accuracy}=\frac{\mathrm{Y}\mathrm{E}\mathrm{S}/\mathrm{N}\mathrm{O}\:\mathrm{c}\mathrm{o}\mathrm{r}\mathrm{r}\mathrm{e}\mathrm{c}\mathrm{t}\:+\:\mathrm{M}\mathrm{C}\mathrm{Q}\:\mathrm{c}\mathrm{o}\mathrm{r}\mathrm{r}\mathrm{e}\mathrm{c}\mathrm{t}}{420}\end{aligned}$$

Because each format contributed 210 responses (30 questions × 7 days), both binary formats were equally weighted.

Open-Ended Weighted Score applies a partial-credit framework to reflect clinically meaningful levels of guideline adherence:

$$\begin{aligned}&\:\text{Open-ended weighted score}=\frac{\begin{aligned}(\boldsymbol{N}.HQ\times\:\:1.0)\:+\left(\boldsymbol{N}.MQ\times\:0.7\right)&+\left(\boldsymbol{N}.LQ\times\:0.3\right)\end{aligned}}{210}\end{aligned}$$

where N denotes the number of responses within each quality category, weighting thresholds were predefined before testing: high quality/full adherence = 1.0; moderate quality/primary recommendation present but secondary details omitted = 0.7; and low quality/primary recommendation absent, guideline-discordant = 0.3. Sensitivity analyses demonstrated that model rankings remained stable across alternative weighting schemes, including strict (1.0/0.5/0.0), lenient (1.0/0.8/0.5), and binary (1.0/0.0/0.0) scoring approaches (Supplementary Table 1). These outcomes were analyzed and reported separately because they assess distinct dimensions of model performance: factual retrieval accuracy for binary questions and guideline-based clinical reasoning quality for open-ended responses.

### Statistical analysis

Statistical analyses were conducted using R (v4.5.0) and SPSS (v23.0). Results for YES/NO and multiple-choice questions were reported as proportions with 95% confidence intervals. Pearson’s chi-square tests assessed associations between LLM platforms and accuracy, as well as differences in the distributions of open-ended response quality across platforms, consistent with established LLM benchmarking methods [14, 26]. Because identical questions were repeated across seven days, responses were not fully independent; however, findings remained robust, with all cross-platform comparisons showing *p* < 0.001 and all temporal stability analyses showing *p* > 0.05. Additional chi-square tests evaluated performance consistency across days for each model. All tests were two-sided, with statistical significance set at *p* < 0.05.

This research did not include human or animal participants and used publicly available AI interfaces; hence, institutional review board approval was unnecessary.

## Results

### Overall response accuracy and quality

A total of 90 clinical questions were posed across three formats (YES/NO, multiple-choice, and open-ended), with each question administered once daily over seven consecutive days on six large language models (LLMs) platforms, yielding 3,780 responses. Of these, 2,520 responses from the YES/NO and multiple-choice questions were categorized as 2,365 correct and 155 incorrect. The remaining 1,260 responses, corresponding to the open-ended questions, were evaluated for quality: 1,048 were classified as high quality, 194 as moderate quality, and 18 as low quality. Performance analysis evaluated correctness by query type, large language models (LLMs)-specific performance across various formats, temporal trends in response accuracy, and binary accuracy and open-ended weighted score metrics, including confidence intervals and statistical significance.

Accuracy varied considerably depending on the query format. For YES/NO queries, 1,204 out of 1,260 responses (95.6%) were accurate, while 56 responses (4.4%) were inaccurate. Similarly, the multiple-choice questions resulted in 1,161 correct responses (92.1%) and 99 incorrect responses (7.9%). In contrast, open-ended queries were assessed according to quality rather than binary correctness: out of 1,260 responses, 1,048 (83.2%) were categorized as high quality, 194 (15.4%) as moderate quality, and 18 (1.4%) as low quality (Table 1).

**Table 1:** Overall response distribution based on question format

TYPE OF QUESTIONS	CORRECTNESS					Total
	CORRECT	INCORRECT	HQ	MQ	LQ	
YES\NO	1204	56	0	0	0	1260
MCQ	1161	99	0	0	0	1260
OPEN ENDED	0	0	1048	194	18	1260
Total	2365	155	1048	194	18	3780

### Large language models (LLMs) performance by question format

**Fig. 2:**
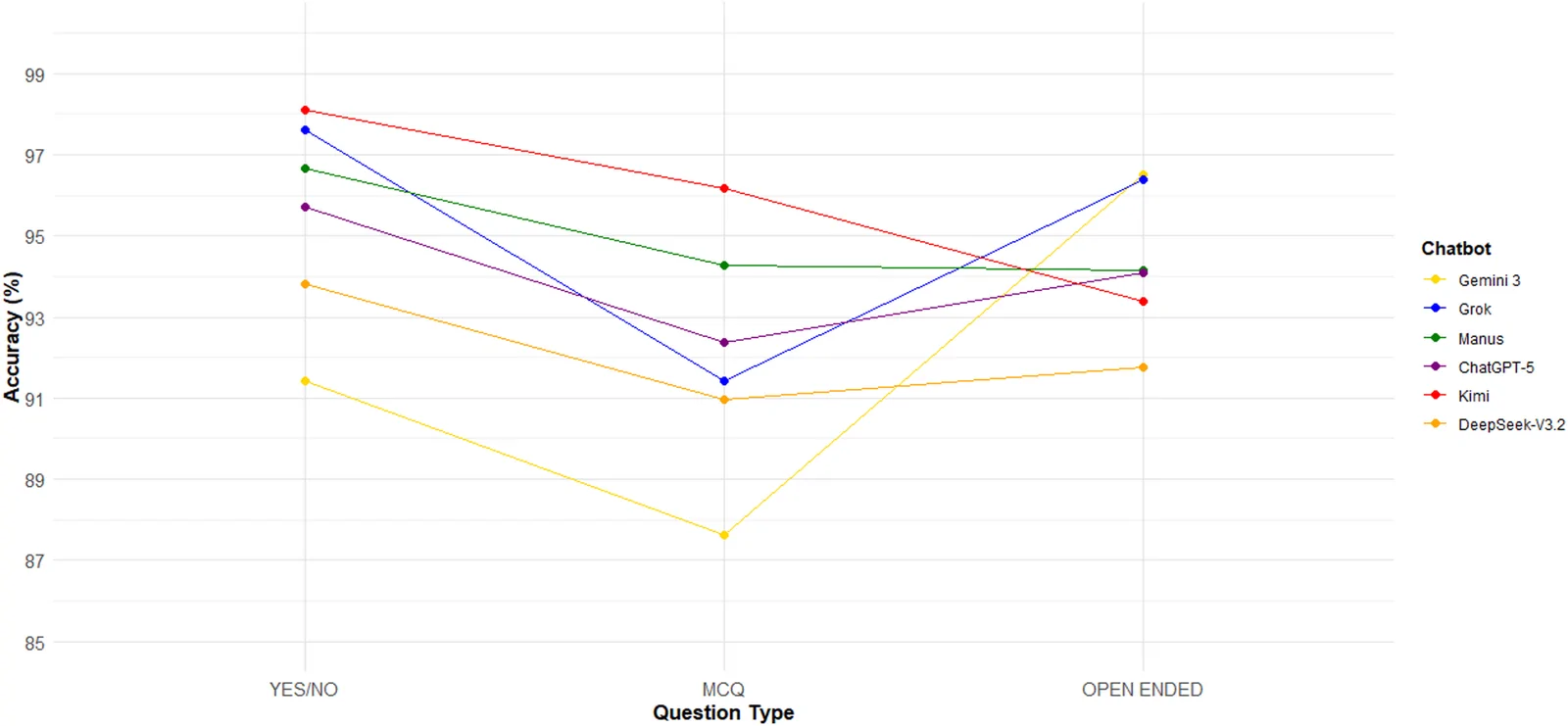
Illustrates the performance distribution of the six large language models across the three question formats. Kimi K1.5 (red) demonstrated the highest accuracy in both YES/NO and MCQ question formats, while Gemini 3 (yellow) achieved the highest accuracy in open-ended questions

Large language models (LLMs) exhibited notable variation in performance across different query formats (*p* < 0.001 for all comparisons). For YES/NO queries, accuracy varied from 91.4% (192/210; 95% CI, 86.9–94.5%) for Gemini 3 to 98.1% (206/210; 95% CI, 95.2–99.3%) for Kimi K1.5. Multiple-choice question accuracy varied from 87.6% (184/210; 95% CI, 82.5–91.4%) for Gemini 3 to 96.2% (202/210; 95% CI, 92.7–98.1%) for Kimi K1.5. In the open-ended queries, the proportion of high-quality responses differed among large language models (LLMs), ranging from 73.8% (155/210) for DeepSeek-V3.2 to 89.0% (187/210) for Gemini 3. When Open-Ended Weighted Scores were utilized for open-ended responses, the average Open-Ended Weighted Score ranged from 91.8% (95% CI, 87.4–94.9%) for DeepSeek-V3.2 to 96.5% (95% CI, 93.3–98.4%) for Gemini 3, with statistically significant differences detected among the models (*p* < 0.001) (Table 2; Fig. 2).

**Table 2:** Model-specific performance by question format

Type of Question	Answers	Gemini 3	Grok	Manus	ChatGPT-5	Kimi K1.5	DeepSeek-V3.2	*P* Value*
YES \NO	correct	192 (91.4%),95% CI: 86.9–94.5%	205 (97.6%),95% CI: 94.6–99.0%	203 (96.7%),95% CI: 93.3–98.4%	201 (95.7%),95% CI: 92.1–97.7%	206 (98.1%),95% CI: 95.2–99.3%	197 (93.8%),95% CI: 89.7–96.4%	< 0.001
	incorrect	18 (8.6%)	5 (2.4%)	7 (3.3%)	9 (4.3%)	4 (1.9%)	13 (6.2%)	
MCQ	correct	184 (87.6%),95% CI: 82.5–91.4%	192 (91.4%),95% CI: 86.9–94.5%	198 (94.3%),95% CI: 90.3–96.7%	194 (92.4%),95% CI: 88.0–95.3%	202 (96.2%),95% CI: 92.7–98.1%	191 (91.0%)95% CI: 86.3–94.1%	< 0.001
	incorrect	26 (12.4%)	18 (8.6%)	12 (5.7%)	16(7.6%)	8 (3.8%)	19 (9.0%)	
OPEN ENDED	high quality	187 (89.0%)	186 (88.6%)	177 (84.3%)	174 (82.9%)	169 (80.5%)	155 (73.8%)	< 0.001
	moderate quality	22 (10.5%)	23 (11.0%)	27 (12.9%)	32 (15.2%)	37 (17.6%)	53 (25.2%)	
	low quality	1 (0.5%)	1 (0.5%)	6 (2.9%)	4 (1.9%)	4 (1.9%)	2 (1.0%)	
	Weighted Score	202.7 (96.5%),	202.4 (96.4%),	197.7 (94.1%),	197.6 (94.1%),	196.1 (93.4%),	192.7 (91.8%),	

*Pearson Chi-Square test

### Longitudinal stability of individual models

When evaluated longitudinally, both binary accuracy and open-ended response quality remained stable across the seven study days for all models, with no apparent day-to-day trend in performance observed across the 7-day study period (all p values > 0.05). During seven days, the total number of correct responses to binary questions ranged from 376 for Gemini 3 to 408 for Kimi K1.5. The incidence of erroneous responses was comparably low for all large language models (LLMs), with Kimi K1.5 documenting 12 inaccuracies and Gemini 3 documenting 44. For open-ended queries, each large language model (LLMs) demonstrated consistently uniform distributions of high, moderate, and low-quality responses throughout the days. High-quality response totals ranged from 155 for DeepSeek-V3.2 to 187 for Gemini 3, while low-quality responses remained infrequent across all models, with totals ranging from 1 to 6 over the entire study period (Table 3).

**Table 3:** Daily performance consistency by model

(LLMs)	Result	Day 1	Day 2	Day 3	Day 4	Day 5	Day 6	Day 7	total	*P* value*
ChatGPT-5	Correct	57	58	55	57	58	56	54	395	0.665
	Incorrect	3	2	5	3	2	4	6	25	
DeepSeek-V3.2	Correct	56	54	57	57	54	55	55	388	0.890
	Incorrect	4	6	3	3	6	5	5	32	
Gemini 3	Correct	53	56	54	53	54	55	51	376	0.840
	Incorrect	7	4	6	7	6	5	9	44	
Grok	Correct	56	58	56	56	57	57	57	397	0.981
	Incorrect	4	2	4	4	3	3	3	23	
Manus	Correct	56	58	58	58	58	56	57	401	0.911
	Incorrect	4	2	2	2	2	4	3	19	
Kimi K1.5	Correct	59	57	59	58	57	60	58	408	0.615
	Incorrect	1	3	1	2	3	0	2	12	
ChatGPT-5	High Quality	24	25	24	25	23	26	27	174	0.698
	Moderate Quality	4	4	5	5	7	4	3	32	
	Low Quality	2	1	1	0	0	0	0	4	
DeepSeek-V3.2	High Quality	22	23	21	22	24	20	23	155	0.837
	Moderate Quality	7	6	9	8	6	10	7	53	
	Low Quality	1	1	0	0	0	0	0	2	
Gemini 3	High Quality	25	28	27	25	27	28	27	187	0.742
	Moderate Quality	4	2	3	5	3	2	3	22	
	Low Quality	1	0	0	0	0	0	0	1	
Grok	High Quality	27	28	24	27	26	26	28	186	0.728
	Moderate Quality	3	2	5	3	4	4	2	23	
	Low Quality	0	0	1	0	0	0	0	1	
Manus	High Quality	20	27	27	25	27	26	25	177	0.278
	Moderate Quality	7	2	3	4	2	4	5	27	
	Low Quality	3	1	0	1	1	0	0	6	
Kimi K1.5	High Quality	22	24	22	26	25	25	25	169	0.196
	Moderate Quality	5	5	8	4	5	5	5	37	
	Low Quality	3	1	0	0	0	0	0	4	

*Pearson Chi-Square test

### A. Binary accuracy of large language models (LLMs)

**Fig. 3:**
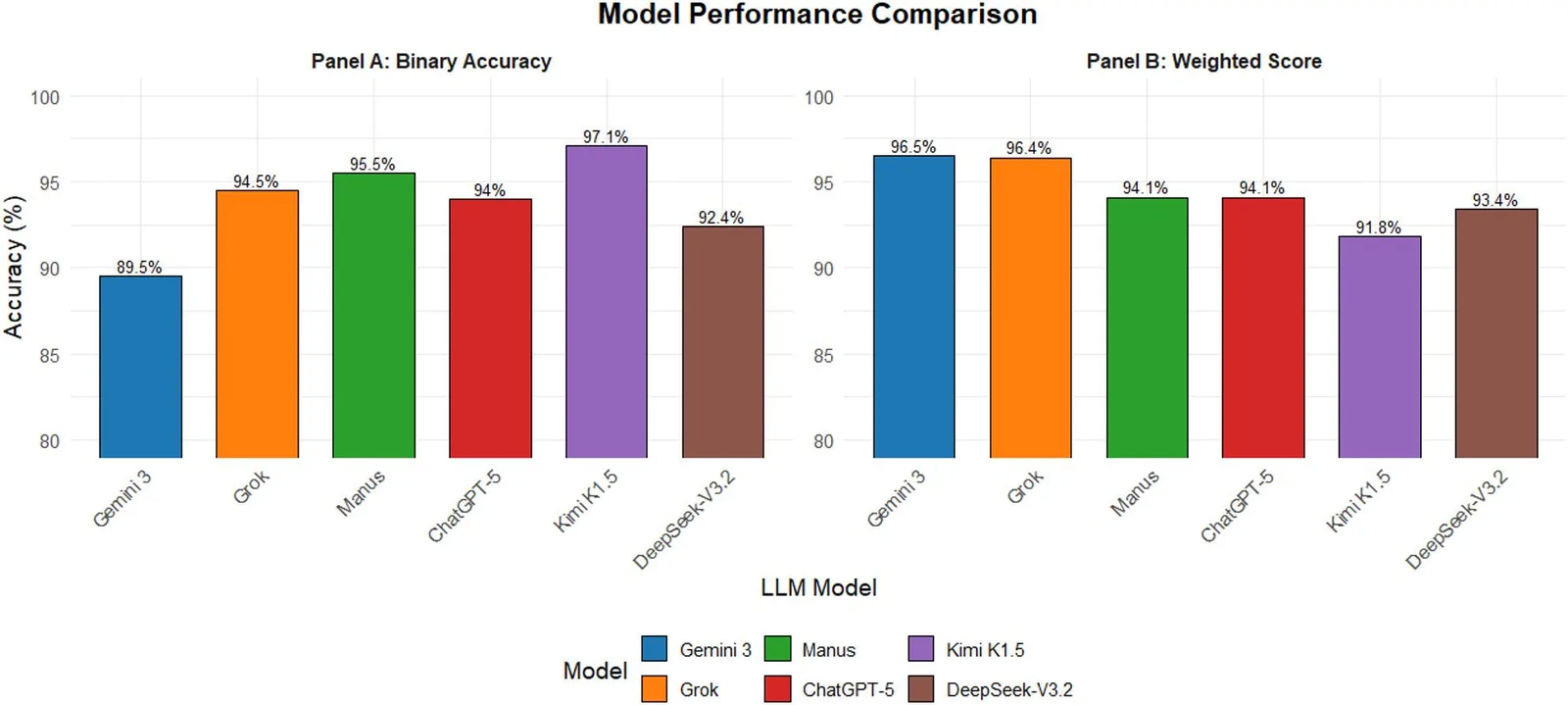
**A**. Binary accuracy (YES/NO + MCQ) of each large language model, **B**. Open-ended weighted score of each large language model.

Binary accuracy (YES/NO and MCQ combined) differed among the six models. Kimi K1.5 demonstrated the highest binary accuracy at 97.1% (408/420; 95% CI, 95.1–98.4%), followed by Manus at 95.5% (401/420; 95% CI, 93.0–97.1%), Grok at 94.5% (397/420; 95% CI, 91.9–96.3%), and ChatGPT-5 at 94.0% (395/420; 95% CI, 91.4–95.9%). DeepSeek-V3.2 achieved 92.4% (388/420; 95% CI, 89.4–94.6%), while Gemini 3 demonstrated the lowest binary accuracy at 89.5% (376/420; 95% CI, 86.2–92.1%) (Table 4A, Fig. 3A).

**Table 4A:** Binary accuracy

Large Language Model	Binary Correct (*n*/420)	Binary Accuracy (%)	95% CI
Kimi K1.5	408	97.1	95.1–98.4
Manus	401	95.5	93.0–97.1
Grok	397	94.5	91.9–96.3
ChatGPT-5	395	94.0	91.4–95.9
DeepSeek-V3.2	388	92.4	89.4–94.6
Gemini 3	376	89.5	86.2–92.1

Table 4A. Binary accuracy (YES/NO and MCQ combined) by model, ranked from highest to lowest

### B. Open-ended weighted score of large language models (LLMs)

Open-Ended Weighted Scores differed among the six models, and the ranking pattern showed partial inversion relative to binary accuracy among the top- and bottom-performing models. Gemini 3 achieved the highest open-ended weighted score at 96.5% (202.7/210; 95% CI, 93.3–98.4%), with 89.0% of its responses classified as high quality. Grok followed at 96.4% (202.4/210; 95% CI, 92.7–98.1%). Manus and ChatGPT-5 each achieved 94.1% (197.7/210 and 197.6/210, respectively; 95% CI, 90.3–96.7%), while Kimi K1.5, the top binary performer, ranked fifth in this domain at 93.4% (196.1/210; 95% CI, 89.1–96.0%). DeepSeek-V3.2 demonstrated the lowest open-ended weighted score at 91.8% (192.7/210; 95% CI, 87.4–94.9%), with only 73.8% of its responses classified as high quality (Table 4B, Fig. 3B).

**Table 4B:** Open-ended weighted score

Large Language Model	Weighted Score (*n*/210)	Weighted Score (%)	95% CI	HQ (%)	MQ (%)	LQ (%)
Gemini 3	202.7	96.5	93.3–98.4	89.0	10.5	0.5
Grok	202.4	96.4	92.7–98.1	88.6	11.0	0.5
Manus	197.7	94.1	90.3–96.7	84.3	12.9	2.9
ChatGPT-5	197.6	94.1	90.3–96.7	82.9	15.2	1.9
Kimi K1.5	196.1	93.4	89.1–96.0	80.5	17.6	1.9
DeepSeek-V3.2	192.7	91.8	87.4–94.9	73.8	25.2	1.0

Table 4B. Open-ended weighted score by model, ranked from highest to lowest. HQ = high quality; MQ = moderate quality; LQ = low quality

Table 5 provides an example of the YES/NO question format and illustrates differences in responses among large language models (LLMs) when identical YES/NO questions were submitted to all platforms on the same day, demonstrating variability in binary responses across models.

**Table 5:** Example YES/NO responses across LLM platforms (same day)

Question	Agreement (YES)	Disagreement (NO)
Does the 2025 GOLD report state that occupational exposures account for 10–20% of symptoms or functional impairment consistent with COPD?	ChatGPT-5, Gemini 3, Manus, Kimi K1.5	DeepSeek-V3.2, Grok

## Discussion

Artificial intelligence (AI) is increasingly transforming clinical practice by improving efficiency, data analysis, and diagnostic support, but it is intended to complement rather than replace clinicians, as human judgment remains essential [27–30]. Despite their utility, AI systems lack the capacity for complex clinical reasoning, highlighting the need for integrated workflows with continuous human oversight [31]. Safe implementation requires multidisciplinary collaboration and rigorous evaluation by professional bodies to ensure reliability, particularly for clinically relevant outputs [32–34]. Although multimodal large language models (M-LLMs) show promise in respiratory medicine, their adoption is limited by challenges in validation, interpretability, integration, and regulation, with emerging governance frameworks (e.g., FDA oversight) reinforcing the need for clinician supervision [35–41].

This study evaluated six advanced large language models (LLMs), ChatGPT-5, DeepSeek-V3.2, Gemini 3, Grok, Manus, and Kimi K1.5, for their responses to the 2025 Global Initiative for Chronic Obstructive Lung Disease (GOLD) guidelines on chronic obstructive pulmonary disease (COPD). These platforms were selected for their widespread accessibility, growing use in healthcare-related information retrieval, and increasing relevance to clinical decision-support research [42, 43]. In addition to assessing response accuracy, the study examined temporal consistency and repeatability across repeated testing sessions. Given the absence of universally accepted performance thresholds for clinical AI systems, this study also aimed to contextualize model performance against the high reliability standards generally expected for tools intended to support clinical decision-making, where prior research has demonstrated substantial variability across models and tasks [44].

Performance across both predefined outcome domains was generally strong but was substantially influenced by question format. Kimi K1.5, Grok, and Manus performed best on objective formats (YES/NO and multiple-choice), whereas Gemini 3, despite lower objective accuracy, received the highest quality ratings for open-ended responses (Fig. 2). This highlights a divergence in current LLM design: some models appear optimized for information retrieval and precise option selection, while others are better suited to generative reasoning and elaborated clinical explanations. These results align with previous assessments demonstrating the high accuracy of large language models on medical multiple-choice examinations, with numerous systems surpassing 90% accuracy and some reaching approximately 95% [45]. Furthermore, systematic reviews have shown that performance differs according to question type, with specific models achieving superior results on objective tasks while others excel in free-text responses, thereby corroborating the format-dependent variability observed in this study [46].

A key finding was the trade-off between binary factual accuracy and open-ended clinical reasoning quality. Kimi K1.5 achieved the highest YES/NO and MCQ accuracy, reflecting strong factual recall and structured data processing, whereas Gemini 3 showed lower binary accuracy but the highest proportion of high-quality open-ended responses and weighted scores. DeepSeek-V3.2 and Kimi K1.5 performed well on forced-choice formats but generated fewer high-quality open-ended responses. These findings suggest that different LLMs may be optimized for different clinical tasks and that multiple-choice accuracy alone may overestimate real-world clinical utility. Prior benchmarking studies similarly report substantially better performance on structured MCQs than on free-response tasks, along with marked variability across models in open-ended clinical reasoning and scenario-generation performance [47, 48].

Performance patterns generally aligned with the reported architectures of the evaluated models. Kimi K1.5 achieved the highest binary accuracy but lower open-ended quality, whereas Gemini 3 showed the strongest open-ended performance despite lower binary accuracy, suggesting stronger contextual synthesis. DeepSeek-V3.2 had the lowest high-quality open-ended response rate, while Grok and ChatGPT-5 demonstrated relatively balanced performance. Manus, evaluated under single-turn constraints, showed strong binary accuracy but the highest proportion of low-quality open-ended responses, possibly reflecting limitations of agent-oriented architectures in isolated clinical reasoning tasks.

Long-term reliability is essential for potential healthcare applications of large language models (LLMs). In this 7-day longitudinal assessment, all models demonstrated stable performance with no significant day-to-day variation (all *p* > 0.05). Binary-question accuracy ranged from 376 correct responses for Gemini 3 to 408 for Kimi K1.5, with consistently low error counts across platforms. Open-ended response quality also remained stable, with high-quality responses ranging from 155 for DeepSeek-V3.2 to 187 for Gemini 3, while low-quality responses were infrequent (1–6 total across the study period) (Table 3). Overall, our results show a high degree of short-term consistency in both the accuracy and quality of responses across various LLM platforms. These findings are consistent with prior studies showing that repeated LLM queries can yield reproducible outputs over time, with newer models such as GPT-4 demonstrating improved response consistency across repeated medical examination questions [49].

Across both primary outcomes, a consistent performance inversion was observed: Kimi K1.5 led in binary accuracy (97.1%) but ranked fifth in open-ended weighted score (93.4%), while Gemini 3 ranked last in binary accuracy (89.5%) yet first in open-ended weighted score (96.5%). This divergence confirms that no single model dominates across all evaluation dimensions. Importantly, none of the evaluated models generated responses that could be considered immediately dangerous or life-threatening, although some responses lacked completeness or precise alignment with guideline recommendations. During the evaluation process, none of the large language models generated responses declining to provide medical guidance (e.g., statements such as “I am an AI and cannot provide medical advice”). Consequently, all responses were evaluated using the predefined scoring rubric based on adherence to the 2025 GOLD guideline recommendations. In medicine, even small mistakes can have big effects. Moreover, the distinction between binary factual correctness and qualitative clinical reasoning underscores the need for multidimensional evaluation frameworks to evaluate these technologies in healthcare applications. Until such frameworks are reliably established, AI large language models (LLMs) need to be used with appropriate clinical supervision rather than as separate decision-making systems.

A primary limitation of this study is that a single researcher conducted both question administration and LLM response evaluation. Although this may introduce observer bias, it was partially mitigated using a structured, percentage-based scoring rubric derived from the 2025 GOLD Report, with independent verification by a senior pulmonary consultant (L.G.). However, the absence of multiple blinded raters and formal inter-rater reliability testing (e.g., Cohen’s kappa) limits the objectivity and reproducibility of qualitative assessments. An additional endogenous limitation is that LLM platforms are not specifically trained on the 2025 GOLD Report; thus, responses may reflect general COPD knowledge from earlier literature rather than explicit incorporation of the most recent guideline updates. From an exogenous perspective, the study was restricted to COPD, a single guideline document, and an English-only evaluation, limiting generalizability to other diseases, clinical guidelines, and multilingual settings. Prompt standardization, while necessary for comparability, may also reduce ecological validity and does not fully reflect real-world clinical complexity, where patient presentations are heterogeneous, and multimorbidity is common. In addition, LLM outputs may be sensitive to prompt wording despite standardization. From a design standpoint, the use of identical question sets over 7 days may limit ecological validity and potentially inflate temporal stability. Furthermore, the relatively short evaluation period may also not capture longer-term variability or model drift. Collectively, these limitations highlight the gap between current LLM performance and the level of robustness required for independent clinical decision-making. Future studies should adopt a coordinated, reproducible evaluation framework featuring: multi-disease and multi-guideline generalizability; API-based model access (temperature = 0, fixed seeds); pre-registered multi-rater validation (weighted Cohen’s kappa); dynamic vignettes plus static questions; longitudinal version tracking; and repeated-measures statistics (e.g., GEE or mixed-effects logistic regression) for within-question clustering.

## Conclusion

This study demonstrates that prominent large language models achieve high but divergent performance across two methodologically distinct outcome domains: binary factual accuracy and open-ended clinical reasoning quality. Both domains demonstrated stable performance across the seven-day evaluation period. However, our research reveals a significant ‘specialization gap’: models such as Kimi K1.5, Grok, and Manus excel as high-precision factual retrieval engines for structured inquiries, whereas Gemini 3 excels at the nuanced clinical reasoning required for complex, open-ended responses. Therefore, while AI large language models (LLMs) can adhere to complex clinical frameworks, their application in COPD care must be tailored to the specific clinical task and remain conditional on expert clinical oversight. Given the remaining risk of factual errors and hallucinations, these tools should remain subordinate to professional clinical judgment and be used as complementary supports rather than standalone decision-making systems.

## Supplementary Information

Below is the link to the electronic supplementary material.

Supplementary Material 1

Supplementary Material 2

Supplementary Material 3

Supplementary Material 4

## Data Availability

The dataset of 90 COPD guideline-based questions used in this study will be made available upon reasonable request and may be released as an open benchmarking resource for future studies evaluating LLM performance in respiratory medicine.

## References

[1] Boers E, et al. Global Burden of Chronic Obstructive Pulmonary Disease Through 2050. JAMA Netw Open. 2023;6(12):e2346598. 38060225 10.1001/jamanetworkopen.2023.46598 PMC10704283

[2] Devine JF. Chronic obstructive pulmonary disease: an overview. Am Health Drug Benefits. 2008;1(7):34–42. 25126252 PMC4106574

[3] Mirza S, et al. COPD Guidelines: A Review of the 2018 GOLD Report. Mayo Clin Proc. 2018;93(10):1488–502. 30286833 10.1016/j.mayocp.2018.05.026

[4] Brusasco V, Martinez F. Chronic obstructive pulmonary disease. Compr Physiol. 2014;4(1):1–31. 24692133 10.1002/cphy.c110037

[5] Xie C, et al. Toward precision medicine in COPD: phenotypes, endotypes, biomarkers, and treatable traits. Respir Res. 2025;26(1):274. 41024111 10.1186/s12931-025-03356-w PMC12482825

[6] Barker BL, Brightling CE. Phenotyping the heterogeneity of chronic obstructive pulmonary disease. Clin Sci (Lond). 2013;124(6):371–87. 23190267 10.1042/CS20120340

[7] Fortis S, et al. Chronic obstructive pulmonary disease (COPD) and COPD-like phenotypes. Front Med (Lausanne). 2024;11:1375457. 38654838 10.3389/fmed.2024.1375457 PMC11037247

[8] Duszyk K, et al. The use of treatable traits to address COPD complexity and heterogeneity and to inform the care. Breathe (Sheff). 2021;17(4):210118. 35035572 10.1183/20734735.0118-2021 PMC8753613

[9] Bartziokas K, et al. Global initiative for chronic obstructive lung disease (GOLD) recommendations: strengths and concerns for future needs. Postgrad Med. 2023;135(4):327–33. 36226501 10.1080/00325481.2022.2135893

[10] (GOLD), G.I.f.C.O.L.D., Global strategy for prevention, diagnosis and management of COPD: 2025 Report. 2024 (official release year).

[11] Shool S, et al. A systematic review of large language model (LLM) evaluations in clinical medicine. BMC Med Inf Decis Mak. 2025;25(1):117. 10.1186/s12911-025-02954-4 PMC11889796 40055694

[12] Pan A, et al. Assessment of Artificial Intelligence Chatbot Responses to Top Searched Queries About Cancer. JAMA Oncol. 2023;9(10):1437–40. 37615960 10.1001/jamaoncol.2023.2947 PMC10450581

[13] Ayers JW, et al. Comparing Physician and Artificial Intelligence Chatbot Responses to Patient Questions Posted to a Public Social Media Forum. JAMA Intern Med. 2023;183(6):589–96. 37115527 10.1001/jamainternmed.2023.1838 PMC10148230

[14] Ekmekci E, Durmazpinar PM. Evaluation of different artificial intelligence applications in responding to regenerative endodontic procedures. BMC Oral Health. 2025;25(1):53. 39799304 10.1186/s12903-025-05424-5 PMC11725189

[15] Shukla M, et al. Assessing the Capabilities of Artificial Intelligence (AI) Tools in Community Medicine: A Comparative Study of ChatGPT, Gemini, and Bing in Community-Based Clinico-Social Case Interpretation. Cureus. 2025;17(9):e91917. 41080342 10.7759/cureus.91917 PMC12510392

[16] Kutbi D, Abou-Bakr E, Haidar HM. Evaluating the Accuracy of Medical Information Generated by ChatGPT and Gemini and Its Alignment With International Clinical Guidelines From the Surviving Sepsis Campaign: Comparative Study. JMIR Form Res. 2025;9:e84251. 41406470 10.2196/84251 PMC12756662

[17] Huang Y, et al. Application of Large Language Models in Complex Clinical Cases: Cross-Sectional Evaluation Study. JMIR Med Inf. 2025;13:e73941. 10.2196/73941 PMC12501899 41055081

[18] Vrdoljak J, et al. A review of large language models in medical education, clinical decision support, and healthcare administration. Healthc (Basel). 2025;13(6). 10.3390/healthcare13060603 PMC11942098 40150453

[19] Pinheira A, et al. Artificial intelligence applications in chronic obstructive pulmonary disease: a global scoping review of diagnostic, symptom-based, and outcome prediction approaches. Biomedicines. 2025;13(12). 10.3390/biomedicines13123053 PMC12730734 41463064

[20] Li D, et al. Streamlining evidence based clinical recommendations with large language models. NPJ Digit Med. 2025;8(1):793. 41423701 10.1038/s41746-025-02273-y PMC12749395

[21] Merç P, Pirinççi C, Cihan E. Evaluation of AI chatbots for patient education and information on chronic obstructive pulmonary disease. Heart Lung. 2026;75:21–5. 40939398 10.1016/j.hrtlng.2025.09.002

[22] Jung KH. Large Language Models in Medicine: Clinical Applications, Technical Challenges, and Ethical Considerations. Healthc Inf Res. 2025;31(2):114–24. 10.4258/hir.2025.31.2.114 PMC12086438 40384063

[23] Roustan D, Bastardot F. The Clinicians’ Guide to Large Language Models: A General Perspective With a Focus on Hallucinations. Interact J Med Res. 2025;14:e59823. 39874574 10.2196/59823 PMC11815294

[24] Kassab J, et al. Accuracy of Online Artificial Intelligence Models in Primary Care Settings. Am J Prev Med. 2024;66(6):1054–9. 38354991 10.1016/j.amepre.2024.02.006

[25] Cavnar Helvaci B, et al. Assessing the accuracy and reliability of ChatGPT’s medical responses about thyroid cancer. Int J Med Inf. 2024;191:105593. 10.1016/j.ijmedinf.2024.105593 39151245

[26] Bashah A, et al. Evaluation of deepseek, gemini, ChatGPT-4o, and perplexity in responding to salivary gland cancer. BMC Oral Health. 2025;25(1):1358. 40849657 10.1186/s12903-025-06726-4 PMC12374294

[27] Maleki Varnosfaderani S, Forouzanfar M. The role of AI in hospitals and clinics: transforming healthcare in the 21st century. Bioeng (Basel). 2024;11(4). 10.3390/bioengineering11040337 PMC11047988 38671759

[28] Bekbolatova M, et al. Transformative potential of AI in healthcare: definitions, applications, and navigating the ethical landscape and public perspectives. Healthc (Basel). 2024;12(2). 10.3390/healthcare12020125 PMC10815906 38255014

[29] Fahim YA, et al. Artificial intelligence in healthcare and medicine: clinical applications, therapeutic advances, and future perspectives. Eur J Med Res. 2025;30(1):848. 40988064 10.1186/s40001-025-03196-w PMC12455834

[30] Sezgin E. Artificial intelligence in healthcare: Complementing, not replacing, doctors and healthcare providers. Digit HEALTH. 2023;9:20552076231186520. 37426593 10.1177/20552076231186520 PMC10328041

[31] Monajemi A. Why is the idea of AI completely replacing physicians a pseudo-problem? a philosophical analysis. J Med Ethics Hist Med. 2025;18:1. 41018553 10.18502/jmehm.v18i1.18814 PMC12474520

[32] Xu L, et al. Chatbot for Health Care and Oncology Applications Using Artificial Intelligence and Machine Learning: Systematic Review. JMIR Cancer. 2021;7(4):e27850. 34847056 10.2196/27850 PMC8669585

[33] Singh K, Prabhu A, Kaur N. The impact and role of artificial intelligence (AI) in healthcare: systematic review. Curr Top Med Chem. 2025. 10.2174/0115680266339394250225112747 40033599

[34] Clerici CA, Chopard S, Levi G. [Rare disease in the age of artificial intelligence]. Recenti Prog Med. 2024;115(2):67–75. 38291931 10.1701/4197.41839

[35] Meskó B. The Impact of Multimodal Large Language Models on Health Care’s Future. J Med Internet Res. 2023;25:e52865. 37917126 10.2196/52865 PMC10654899

[36] AlSaad R, et al. Multimodal Large Language Models in Health Care: Applications, Challenges, and Future Outlook. J Med Internet Res. 2024;26:e59505. 39321458 10.2196/59505 PMC11464944

[37] Yang X, et al. Application of large language models in disease diagnosis and treatment. Chin Med J (Engl). 2025;138(2):130–42. 39722188 10.1097/CM9.0000000000003456 PMC11745858

[38] Meng X, et al. The application of large language models in medicine: A scoping review. iScience. 2024;27(5):109713. 38746668 10.1016/j.isci.2024.109713 PMC11091685

[39] Shumway DO, Hartman HJ. Medical malpractice liability in large language model artificial intelligence: legal review and policy recommendations. J Osteopath Med. 2024;124(7):287–90. 38295300 10.1515/jom-2023-0229

[40] Weissman GE. Evaluation and Regulation of Artificial Intelligence Medical Devices for Clinical Decision Support. Annu Rev Biomed Data Sci. 2025;8(1):81–99. 39971383 10.1146/annurev-biodatasci-103123-095824 PMC12339208

[41] Giorgetti C, Giorgetti A, Boscolo-Berto R. Establishing new boundaries for medical liability: The role of AI as a decision-maker. Adv Clin Exp Med. 2025;34(10):1601–6. 40788001 10.17219/acem/208596

[42] Ozduran E, et al. Assessing the readability, quality and reliability of responses produced by ChatGPT, Gemini, and Perplexity regarding most frequently asked keywords about low back pain. PeerJ. 2025;13:e18847. 39866564 10.7717/peerj.18847 PMC11760201

[43] Aykac K, et al. Comparing ChatGPT-3.5, Gemini 2.0, and DeepSeek V3 for pediatric pneumonia learning in medical students. Sci Rep. 2025;15(1):40342. 41254112 10.1038/s41598-025-27722-2 PMC12627666

[44] Waldock WJ, et al. The Accuracy and Capability of Artificial Intelligence Solutions in Health Care Examinations and Certificates: Systematic Review and Meta-Analysis. J Med Internet Res. 2024;26:e56532. 39499913 10.2196/56532 PMC11576595

[45] Agarwal M, Sharma P, Wani P. Evaluating the Accuracy and Reliability of Large Language Models (ChatGPT, Claude, DeepSeek, Gemini, Grok, and Le Chat) in Answering Item-Analyzed Multiple-Choice Questions on Blood Physiology. Cureus. 2025;17(4):e81871. 40342473 10.7759/cureus.81871 PMC12060195

[46] Wang L, et al. Accuracy of Large Language Models When Answering Clinical Research Questions: Systematic Review and Network Meta-Analysis. J Med Internet Res. 2025;27:e64486. 40305085 10.2196/64486 PMC12079073

[47] Singh S, et al. The pitfalls of multiple-choice questions in generative AI and medical education. Sci Rep. 2025;15(1):42096. 41298584 10.1038/s41598-025-26036-7 PMC12658246

[48] Szabó A, Laein GD. Comparative evaluation of large language models performance in medical education using urinary system histology assessment. Sci Rep. 2025;15(1):31933. 40883524 10.1038/s41598-025-17571-4 PMC12397380

[49] Funk PF, et al. ChatGPT’s Response Consistency: A Study on Repeated Queries of Medical Examination Questions. Eur J Investig Health Psychol Educ. 2024;14(3):657–68. 38534904 10.3390/ejihpe14030043 PMC10969490

