# Supplementary material for: AI in respiratory care: findings from the GOLD report
Source: J Transl Med. 2026 Jun 13;24:1082. doi: 10.1186/s12967-026-08454-x (PMC13501795; doi:10.1186/s12967-026-08454-x)
Supplement: Supplementary file 1 [file 12967_2026_8454_MOESM1_ESM.docx]

**YES \ NO QUESTIONS FORMAT**

Q1: Is COPD now one of the top three leading causes of death worldwide?

Q2: Does the 2025 GOLD report recommend using pre-bronchodilator FEV1/FVC < 0.7 as the mandatory diagnostic criterion for COPD?

Q3: Is the term "early COPD" recommended for use in clinical practice to describe the initial perception of symptoms?

Q4: Is household air pollution from biomass fuels a risk factor for COPD, particularly in low- and middle-income countries?

Q5: Does the 2025 GOLD ABE assessment tool classify patients based on both symptom burden and exacerbation history?

Q6: Is a post-bronchodilator FEV1/FVC ratio between 0.6 and 0.8 considered definitive for diagnosing airflow obstruction without needing repeat spirometry?

Q7: Should all patients with COPD, regardless of symptom level or exacerbation history, be initiated on inhaled corticosteroid (ICS) therapy?

Q7: Should all patients with COPD, regardless of symptom level or exacerbation history, be initiated on inhaled corticosteroid (ICS) therapy?

Q8: Is the COPD Assessment Test (CAT) score of ≥10 equivalent to an SGRQ score of ≥25 for identifying significant symptom burden?

Q9: Does long-term oxygen therapy (LTOT) improve survival in patients with stable COPD and moderate resting or exercise-induced arterial desaturation?

Q10: Is pulmonary rehabilitation recommended only for patients with severe (GOLD 3-4) airflow obstruction?

Q11: Is a blood eosinophil count ≥300 cells/μL a factor to consider when initiating triple therapy (LABA+LAMA+ICS) in a newly diagnosed Group E patient?

Q12: Does the 2025 GOLD report include a dedicated chapter on COVID-19 and COPD?

Q13: Is dysanapsis defined as an anthropometric mismatch of airway tree caliber relative to lung volume?

Q14: Should spirometry be repeated at least annually in patients with COPD for monitoring purposes?

Q15: Is it necessary for patients to stop their regular inhaled bronchodilator medication before performing spirometry for monitoring?

Q16: Is the use of race-neutral GLI-Global reference equations now recommended by GOLD for interpreting lung function?

Q17: Is the presence of chronic bronchitis (chronic cough and sputum) necessary for a diagnosis of COPD?

Q18: Are individuals with "Pre-COPD" or "PRISm" (Preserved Ratio Impaired Spirometry) considered to have COPD?

Q19: Does the 2025 GOLD report recommend the use of e-cigarettes as a smoking cessation aid for patients with COPD?

Q20: Is long-term non-invasive ventilation (NIV) indicated for all patients with stable COPD and severe airflow obstruction?

Q21: Is the fixed FEV1/FVC ratio of <0.7 preferred by GOLD over the Lower Limit of Normal (LLN) for diagnosing airflow obstruction due to diagnostic simplicity?

Q22: Should a patient with COPD who is well-controlled on LABA+ICS therapy (with no features of asthma) be switched immediately to LABA+LAMA?

Q23: Is the Tdap (dTap/dTPa) vaccination recommended for adults with COPD who were not vaccinated in adolescence?

Q24: Is the term "Asthma-COPD Overlap (ACO)" used in the 2025 GOLD report to guide pharmacotherapy?

Q25: Does the 2025 GOLD report state that occupational exposures account for 10-20% of symptoms or functional impairment consistent with COPD?

Q26: Is dynamic hyperinflation primarily a consequence of loss of elastic lung recoil?

Q27: Should a chest X-ray be performed routinely to establish a diagnosis of COPD?

Q28: Is the BODE index a composite score that is a better predictor of survival in COPD than FEV1 alone?

Q29: Does the 2025 GOLD report include a new section on Climate Change & COPD?

Q30: Is screening spirometry recommended for the general asymptomatic population without risk factors?
