# Supplementary material for: AI in respiratory care: findings from the GOLD report
Source: J Transl Med. 2026 Jun 13;24:1082. doi: 10.1186/s12967-026-08454-x (PMC13501795; doi:10.1186/s12967-026-08454-x)
Supplement: Supplementary file 2 [file 12967_2026_8454_MOESM2_ESM.docx]

MULTIPLE CHOICE QUESTIONS FORMAT

Q31: According to GOLD 2025, what is the definitive diagnostic test required to establish a diagnosis of COPD?

A. Chest X-ray

B. Post-bronchodilator spirometry showing FEV1/FVC < 0.7

C. High-resolution CT scan showing emphysema

D. Measurement of blood eosinophil count

Q32: A 65-year-old patient with a 40-pack-year smoking history presents with an mMRC score of 1 and a CAT score of 8. They had one moderate exacerbation treated with oral corticosteroids and antibiotics in the past year. According to the GOLD 2025 ABE assessment tool, how is this patient classified?

A. Group A

B. Group B

C. Group E

D. Cannot be classified

Q33: Which of the following is a key recommended component of initial non-pharmacological management for a patient newly diagnosed with COPD in GOLD Group B?

A. Long-term oxygen therapy

B. Pulmonary rehabilitation

C. No intervention needed

D. Nocturnal non-invasive ventilation

Q34: According to GOLD 2025, what is the recommended initial pharmacological treatment for a patient in Group E (high exacerbation risk) with a blood eosinophil count of 400 cells/μL?

A. LAMA monotherapy

B. LABA monotherapy

C. LABA + LAMA

D. LABA + LAMA + ICS

Q35: For which of the following patients is long-term oxygen therapy (LTOT) unequivocally indicated to improve survival?

A. Stable COPD with PaO2 of 65 mmHg and peripheral edema

B. Stable COPD with PaO2 of 58 mmHg and hematocrit of 56%

C. Stable COPD with exercise-induced desaturation to SpO2 88%

D. Stable COPD with PaO2 of 54 mmHg confirmed twice over three weeks

Q36: A patient with stable COPD is on LABA+LAMA therapy but continues to have persistent dyspnea (mMRC 3). Inhaler technique and adherence are good. According to the 2025 follow-up algorithm, what is the next recommended step?

A. Add ICS

B. Add roflumilast

C. Add theophylline

D. Implement or escalate non-pharmacological treatment (e.g., pulmonary rehabilitation)

Q37: Which of the following is a recommended use of chest CT imaging in stable COPD according to GOLD 2025?

A. Routine diagnosis in all patients

B. Evaluation for lung volume reduction in patients with FEV1 15-45% predicted and hyperinflation

C. Annual screening for pulmonary hypertension

D. To confirm the presence of chronic bronchitis

Q38: What does the PRISm (Preserved Ratio Impaired Spirometry) classification indicate?

A. FEV1/FVC < 0.7 and FEV1 < 80% predicted

B. FEV1/FVC ≥ 0.7 and FEV1 < 80% predicted

C. FEV1/FVC < 0.7 and FEV1 ≥ 80% predicted

D. The presence of emphysema on CT with normal spirometry

Q39: According to the 2025 vaccination recommendations, which vaccine is specifically recommended for individuals with COPD aged ≥60 years?

A. Bacillus Calmette-Guérin (BCG)

B. Respiratory Syncytial Virus (RSV) vaccine

C. Meningococcal vaccine

D. Varicella vaccine

Q40: In the follow-up pharmacological management pathway, a patient on LABA+LAMA therapy continues to have exacerbations. Their blood eosinophil count is 50 cells/μL. Which of the following is a recommended add-on therapy option?

A. Add Inhaled Corticosteroid (ICS)

B. Add Dupilumab

C. Add Azithromycin (if not a current smoker)

D. Add Ensifentrine

Q41: What is the primary mechanism by which hyperinflation contributes to dyspnea in COPD?

A. By increasing pulmonary vascular resistance

B. By placing the respiratory muscles at a mechanical disadvantage

C. By causing metabolic acidosis

D. By reducing cardiac output through tamponade

Q42: Which of the following is a major change in the 2025 GOLD report regarding patient assessment?

A. Reintroduction of the ABCD groups including spirometric grade

B. Replacement of Groups C and D with a single Group E

C. Removal of symptom assessment from the classification

D. Use of exacerbation history only for Group D

Q43: A patient with COPD has a clinical diagnosis of bronchiectasis confirmed on CT. How should this finding influence management?

A. It does not change COPD management

B. It mandates the use of inhaled corticosteroids

C. It should prompt investigation and management according to bronchiectasis guidelines

D. It is an indication for long-term macrolide therapy regardless of exacerbation history

Q44: What is the recommended threshold of blood eosinophil count that makes exacerbations after ICS withdrawal more likely?

A. ≥100 cells/μL

B. ≥150 cells/μL

C. ≥300 cells/μL

D. ≥500 cells/μL

Q45: For a patient with stable COPD, which of the following is a key goal of treatment?

A. Fully reverse airflow obstruction

B. Cure the underlying disease

C. Reduce both symptoms and future risk of adverse events

D. Prevent all exacerbations

**CHOOSE THE BEST ANSWER:**

Q46: What is the recommended first-line pharmacotherapy for a Group B patient (high symptoms, low exacerbation risk)?

A. Short-acting bronchodilator PRN

B. LABA or LAMA monotherapy

C. LABA + LAMA

D. LABA + ICS

Q47: Which of the following is an indication for considering surgical lung volume reduction (LVRS) in emphysema?

A. Any patient with GOLD 4 airflow obstruction

B. Non-upper lobe emphysema with high exercise capacity

C. Upper lobe predominant emphysema with low exercise capacity after rehabilitation

D. Diffuse emphysema with chronic bronchitis

Q48: What is the single most effective strategy to reduce the progression of COPD in a current smoker?

A. Regular use of inhaled corticosteroids

B. Smoking cessation

C. Annual pneumococcal vaccination

D. Participation in pulmonary rehabilitation

Q49: According to GOLD 2025, when should screening for Alpha-1 Antitrypsin Deficiency (AATD) be performed?

A. Only in patients with basilar predominant emphysema

B. Only in patients under age 45

C. Once in all patients diagnosed with COPD

D. Only if there is a family history of liver disease

Q50: In the management of a patient with advanced COPD and refractory dyspnea despite optimal therapy, which intervention has evidence for palliation of breathlessness?

A. Regular benzodiazepines

B. Opioids

C. Supplemental oxygen in non-hypoxemic patients

D. Both B and C

Q51: What is the recommended approach if a patient's pre-bronchodilator spirometry shows FEV1/FVC < 0.7?

A. Diagnose COPD immediately

B. Perform post-bronchodilator spirometry to confirm the diagnosis

C. Initiate long-acting bronchodilator therapy

D. Order a chest CT scan

Q52: Which of the following factors is part of the GETomics concept for COPD pathogenesis?

A. Genetics (G), Environment (E), and Time (T) interactions

B. Gender, Ethnicity, and Tobacco exposure

C. Genotype, Emphysema, and Treatment

D. Growth factors, Epigenetics, and Trauma

Q53: For a patient undergoing lung cancer screening with low-dose CT who is found to have significant emphysema but no prior COPD diagnosis, what is the recommended next step?

A. No action is needed

B. Perform spirometry to assess for airflow obstruction

C. Start empiric treatment with a long-acting bronchodilator

D. Schedule a follow-up CT in one year

Q54: What is the role of the blood eosinophil count in the initial assessment of COPD?

A. To diagnose COPD

B. To guide the use of inhaled corticosteroids for exacerbation prevention

C. To determine eligibility for pulmonary rehabilitation

D. To screen for allergic bronchopulmonary aspergillosis (ABPA)

Q55: Which of the following statements about pulmonary rehabilitation (PR) is TRUE according to GOLD 2025?

A. It is only effective in a hospital-based setting.

B. Telerehabilitation can achieve similar clinical outcomes as center-based PR.

C. Benefits are primarily due to educational sessions.

D. It is contraindicated in patients with cardiovascular comorbidities.

Q56: A patient with COPD and features of asthma (e.g., childhood onset, atopy, large bronchodilator response) should have their pharmacotherapy guided primarily by:

A. COPD guidelines

B. Asthma guidelines

C. Both sets of guidelines equally

D. The presence of eosinophilia only

Q57: Which of the following is a new therapeutic agent added to the 2025 GOLD report figures (e.g., 3.9, 3.18, 3.20)?

A. Tezepelumab

B. Ensifentrine

C. Benralizumab

D. Montelukast

Q58: In the context of climate change, what indoor temperature is recommended for bedrooms during cold weather for people with COPD?

A. Above 15°C

B. Above 18°C

C. Above 21°C

D. Above 24°C

Q59: What is the recommended first step if a patient on follow-up has an inadequate response to initial pharmacological therapy?

A. Immediately escalate pharmacotherapy

B. Check inhaler technique and adherence

C. Order a chest CT scan

D. Refer for lung volume reduction surgery

Q60: Which of the following vaccines is NOT listed in the 2025 GOLD vaccination recommendations for stable COPD?

A. Influenza

B. Pneumococcal (PCV20/PCV21/PPSV23)

C. Respiratory Syncytial Virus (RSV)

D. Hepatitis B
