# Supplementary material for: AI in respiratory care: findings from the GOLD report
Source: J Transl Med. 2026 Jun 13;24:1082. doi: 10.1186/s12967-026-08454-x (PMC13501795; doi:10.1186/s12967-026-08454-x)
Supplement: Supplementary file 3 [file 12967_2026_8454_MOESM3_ESM.docx]

**OPEN ENDED QUESTIONS**

Q61: According to GOLD 2025, what are the two main treatment goals for stable COPD management?

Q62: Briefly describe the GOLD 2025 ABE assessment tool. What two patient characteristics determine the group (A, B, or E)?

Q63: A patient has a post-bronchodilator FEV1 of 45% predicted and FEV1/FVC of 0.65. What is their GOLD grade of airflow obstruction severity?

Q64: What does GOLD 2025 recommend regarding the assessment of "reversibility" (change in FEV1 before and after bronchodilator) for diagnostic or therapeutic decision-making?

Q65: According to the 2025 update, what should be done with a patient currently on LABA+ICS who is well-controlled (no exacerbations, minimal symptoms) and has no features of asthma?

Q66: Name two non-pharmacological interventions that are "Essential" for initial management of a patient in GOLD Group B at diagnosis.

Q67: What are the two key "treatable traits" used to guide follow-up pharmacological management in the 2025 algorithm (Figure 3.9)?

Q68: According to GOLD 2025, what are the two main pathophysiological mechanisms that cause expiratory airflow obstruction in COPD?

Q69: What is the significance of finding "interstitial lung abnormalities (ILA)" on a CT scan in a patient with COPD?

Q70: Describe the recommended step-wise approach for a clinician choosing an inhaler device for a patient with COPD.

Q71: Under what circumstances should withdrawal of inhaled corticosteroids (ICS) be considered in a patient on triple therapy (LABA+LAMA+ICS)?

Q72: How does GOLD 2025 define an exacerbation of COPD?

Q73: What is the role of the blood eosinophil count in guiding the addition of ICS to dual bronchodilator therapy (LABA+LAMA) for exacerbation prevention?

Q74: Name two conditions that are common comorbidities in COPD and can mimic or aggravate an acute exacerbation.?

Q75: What is the proposed taxonomic (etiologic) classification for COPD introduced in Chapter 1, and name two examples of etiotypes.

Q76: What is the primary rationale for GOLD continuing to recommend the fixed FEV1/FVC ratio <0.7 over the Lower Limit of Normal (LLN)?

Q77: According to the 2025 follow-up algorithm, what are the recommended options for a patient on LABA+LAMA+ICS who continues to have exacerbations and has blood eosinophils ≥300 cells/μL with symptoms of chronic bronchitis?

Q78: What does GOLD 2025 state about the relationship between the severity of airflow obstruction (FEV1) and the level of symptoms a patient experiences?

Q79: What is the recommended management approach for "refractory dyspnea" in advanced COPD despite optimal medical therapy?

Q80: Why is it important to consider cardiovascular risk during and after an exacerbation of COPD (ECOPD)?

Q81: What is the role of a "written action plan" in COPD self-management education?

Q82: How has the section on "Withdrawal of ICS" been updated in the 2025 report?

Q83: According to the 2025 report, what is the single best predictor of having frequent exacerbations (≥2 per year)?

Q84: What are the two key components that define "Pre-COPD"?

Q85: What is the minimum recommended duration for an effective pulmonary rehabilitation program?

Q86: Name two new drugs mentioned in the 2025 pharmacotherapy figures (3.9, 3.18, 3.20) that were not in the previous report.

Q87: What does GOLD 2025 recommend regarding case-finding versus screening for COPD?

Q88: Briefly explain why the 2025 report merged the old Groups C and D into a single Group E.

Q89: What is the recommended first-line therapy for a Group A patient (low symptoms, low exacerbation risk)?

Q90: How should a clinician proceed if a patient's pre-bronchodilator FEV1/FVC is ≥0.7, but there is a high clinical suspicion for COPD?
