# Supplementary material for: AI in respiratory care: findings from the GOLD report
Source: J Transl Med. 2026 Jun 13;24:1082. doi: 10.1186/s12967-026-08454-x (PMC13501795; doi:10.1186/s12967-026-08454-x)
Supplement: Supplementary file 4 [file 12967_2026_8454_MOESM4_ESM.docx]

**sensitivity analyses for weighting schemes**

| Model | Primary (1.0/0.7/0.3) | Strict (1.0/0.5/0.0) | Lenient (1.0/0.8/0.5) | Binary (1.0/0.0/0.0) |
| --- | --- | --- | --- | --- |
| Gemini 3 | 96.5% (1st) | 94.3% (1st) | 97.7% (1st) | 89.0% (1st) |
| Grok | 96.4% (2nd) | 94.0% (2nd) | 97.6% (2nd) | 88.6% (2nd) |
| Manus | 94.1% (3rd) | 90.7% (3rd) | 96.0% (3rd) | 84.3% (3rd) |
| ChatGPT‑5 | 94.1% (4th) | 90.5% (4th) | 96.0% (4th) | 82.9% (4th) |
| Kimi K1.5 | 93.4% (5th) | 89.3% (5th) | 95.5% (5th) | 80.5% (5th) |
| DeepSeek‑V3.2 | 91.8% (6th) | 86.4% (6th) | 94.5% (6th) | 73.8% (6th) |
